# High Circulating Methylated DNA Is a Negative Predictive and Prognostic Marker in Metastatic Colorectal Cancer Patients Treated With Regorafenib

**DOI:** 10.3389/fonc.2019.00622

**Published:** 2019-07-12

**Authors:** Alessio Amatu, Marta Schirripa, Federica Tosi, Sara Lonardi, Katia Bencardino, Erica Bonazzina, Laura Palmeri, Damiano Alfio Patanè, Elio Gregory Pizzutilo, Benedetta Mussolin, Francesca Bergamo, Giulia Alberti, Rossana Intini, Letizia Procaccio, Marco Arese, Silvia Marsoni, Michele Nichelatti, Vittorina Zagonel, Salvatore Siena, Alberto Bardelli, Fotios Loupakis, Federica Di Nicolantonio, Andrea Sartore-Bianchi, Ludovic Barault

**Affiliations:** ^1^Niguarda Cancer Center, Grande Ospedale Metropolitano Niguarda, Milan, Italy; ^2^Medical Oncology 1, Veneto Institute of Oncology, IRCCS, Padua, Italy; ^3^Candiolo Cancer Institute, FPO–IRCCS, Candiolo, Italy; ^4^Department of Surgery, Oncology and Gastroenterology, University of Padua, Padua, Italy; ^5^Department of Oncology, University of Turin, Candiolo, Italy; ^6^Department of Precision Oncology, FIRC Institute of Molecular Oncology (IFOM), Milan, Italy; ^7^Dipartimento di Oncologia ed Emato-Oncologia, Università degli Studi di Milano, Milan, Italy

**Keywords:** regorafenib, DNA methylation, metastatic colorectal cancer, cell free circulating DNA, liquid biopsy, digital PCR, biomarkers, prognosis

## Abstract

**Background:** Regorafenib improves progression free survival (PFS) in a subset of metastatic colorectal cancer (mCRC) patients, although no biomarkers of efficacy are available. Circulating methylated DNA (cmDNA) assessed by a five-gene panel was previously associated with outcome in chemotherapy treated mCRC patients. We hypothesized that cmDNA could be used to identify cases most likely to benefit from regorafenib (i.e., patients with PFS longer than 4 months).

**Methods:** Plasma samples from mCRC patients were collected prior to (baseline samples *N* = 60) and/or during regorafenib treatment (*N* = 62) for the assessment of cmDNA and total amount of cell free DNA (cfDNA).

**Results:** In almost all patients, treatment with regorafenib increased the total cfDNA, but decreased cmDNA warranting the normalization of cmDNA to the total amount of circulating DNA (i.e., cmDNA/ml). We report that cmDNA/ml dynamics reflects clinical response with an increase in cmDNA/ml associated with higher risk of progression (HR for progression = 1.78 [95%CI: 1.01–3.13], *p* = 0.028). Taken individually, high baseline cmDNA/ml (above median) was associated with worst prognosis (HR for death = 3.471 [95%CI: 1.83–6.57], *p* < 0.0001) and also predicted shorter PFS (<16 weeks with PPV 86%). In addition, high cmDNA/ml values during regorafenib treatment predicted with higher accuracy shorter PFS (<16 weeks with a PPV of 96%), therefore associated with increased risk of progression (HR for progression = 2.985; [95%CI: 1.63–5.46; *p* < 0.0001).

**Conclusions:** Our data highlight the predictive and prognostic value of cmDNA/ml in mCRC patients treated with regorafenib.

## Background

Colorectal cancer (CRC) remains the third most common cancer in men and women worldwide (1). The advent of new therapeutic agents has enhanced the median overall survival (OS) up to 30 months for metastatic CRC (mCRC) patients (2, 3). Among the new lines of treatment added to the therapeutic armamentarium of mCRC, regorafenib is a multikinase inhibitor targeting angiogenic and oncogenic activities in the tumor and its stroma. It has demonstrated single agent efficacy in preclinical cancer models (4, 5) and in patients with chemo-refractory mCRC (6). In 2013, the phase III CORRECT study (7) showed a median OS improvement of 1.4 months, leading to the approval of regorafenib by the EMA. While mCRC patients treated with regorafenib achieved a response rate of 1% and a 16-week disease control rate in 19% cases, up to 54% individuals experienced grade 3 or 4 treatment related adverse events such as hand-foot skin reaction, fever, and fatigue, which severely impair quality of life (8). Consequently, the overall clinical benefit from regorafenib remains rather limited. While no validated biomarkers are available to prospectively identify individuals who could benefit from this drug, several studies have previously explored the use of circulating biomarkers.

A retrospective exploratory analysis of the CORRECT trial showed that baseline circulating total cell free DNA concentration was prognostic rather than predictive for clinical outcome; since both placebo and regorafenib provided a consistent survival benefit in a subset of patients based on low amount of tumor mutation and plasma protein biomarker concentrations (9). Another retrospective analysis by Komori et al. demonstrated that an early decrease in serum CA19–9 protein levels could predict for regorafenib efficacy and was associated with better progression free survival (PFS) in mCRC (10). In a phase II study, a profound decrease of *RAS* mutant clones in circulating tumor DNA (ctDNA) was associated with better PFS in 21 mCRC patients after 8 weeks of treatment with regorafenib, together with modification at dynamic contrast-enhanced MRI (11). Finally, Vandeputte et al. showed the prognostic value of monitoring genetic alterations in the ctDNA of a small cohort of 20 patients. This approach implied next generation sequencing of patient primary tumors and optimization of personalized assays for mutation tracking in plasma (12).

The above-mentioned circulating biomarker studies in mCRC patients undergoing regorafenib treatment were either based on a single parameter or on panels of genetic alterations requiring expensive and time-consuming personalized assay design. Nevertheless, simpler and more universally applicable biomarker would be desirable to improve cost-effectiveness of regorafenib treatment.

We and others recently demonstrated that cancer specific DNA methylation could represent a promising analyte for circulating tumor markers. Thanks to its stability and its specificity to cancer, CRC epigenetic alterations could be detected in plasma cell free DNA at higher prevalence and with a higher allelic ratio than genetic alterations. Some of these methylated loci were also identified as early events in the carcinogenesis process, representing promising cancer specific tools for early diagnosis using blood tests (13–20).

We previously identified a panel of five methylated genes (*EYA4, GRIA4, ITGA4, MAP3K14-AS1, MSC*) and used it in a liquid biopsy (LB) test to monitor mCRC tumor burden over the course of conventional chemotherapy regimens (21). Yet, the dynamics of ctDNA under treatment with regorafenib remains to be investigated.

Inspired by data of two recent studies (11, 12) with limited number of patients and using exclusively genetic alterations, we hypothesized that dynamics of circulating methylated DNA (cmDNA) may stratify mCRC patients treated with regorafenib according to their clinical outcome sparing a subgroup of unresponsive patients from prolonged drug exposure.

## Patients and Methods

### Patient Selection and Study Design

**Figure 1:**
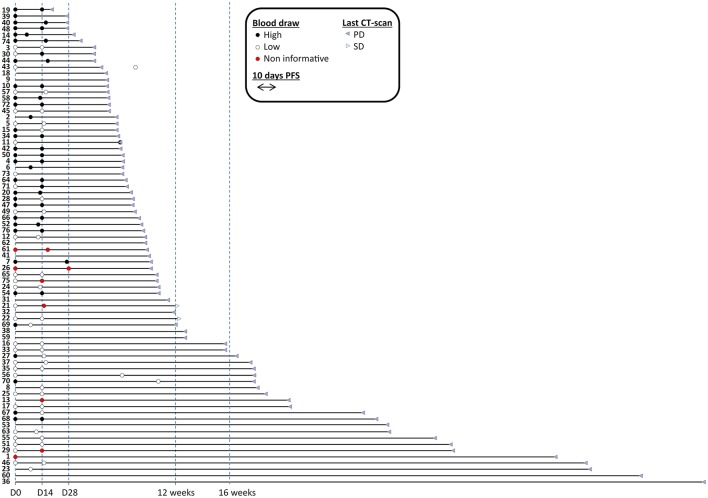
Time to progression of patients enrolled in the study with sample availability and informativeness. Blood draws are represented with filled circles and colored in black when cmDNA/ml was above median, depicted in white when cmDNA/ml was below median; red circles label samples that were not informative (i.e., no positive markers). Patients were sorted by duration of progression free survival. Sixty baseline (among which 57 informative) and 62 under treatment blood draws (among which 56 informative) were available. Fifty seven patients had both types of blood draws available (among which 52 informative in both cases). Two cases were censored for follow-up. Imaging CT-scans are also indicated.

We selected 76 mCRC patients who received regorafenib at Niguarda Cancer Center, NCC (Milano) or Istituto Oncologico Veneto, IOV (Padova) from December 2012 to August 2017 (Supplementary Table S1). LB samples were collected prospectively and analyzed retrospectively in double blind fashion for patient outcome (Figure 1).

Eligible patients had histologically confirmed metastatic adenocarcinoma of the colon or rectum, performance status (PS) 0–1, adequate organ function, age >18 years, life expectancy of at least 12 weeks (based on physician's prognostication according to patient age, ECOG performance status, general conditions, comorbidities, and lab tests including renal and liver function), and were refractory or intolerant to conventional 5-FU-based chemotherapy treatments. Patients received 160 mg of regorafenib orally once daily for the first 3 weeks of each 4-week cycle, with dose reduction according to physician's discretion and current guidelines (Supplementary Tables S1, S2).

Blood was collected at baseline (prior to regorafenib start) and/or then biweekly or at any subsequent access to the hospital, during regorafenib treatment.

All the patients provided written informed consent to LB collection before and during regorafenib treatment. Protocol for blood collection and analysis was approved from NCC and IOV institutional Ethics Committees.

### Assessment of Cell Free Circulating DNA Markers

Analysis of cell free circulating DNA (cfDNA) via a panel of methylated CRC-specific genes was carried out on blood as previously described (21).

We defined a value of cmDNA compiled as the methylation average of the markers demonstrating positivity above the previously published thresholds (21), supported by number of methylated events above the limit of detection. In subsequent longitudinal LB, for each patient, calculation was based exclusively on the loci used to compile the cmDNA in the baseline.

Additionally, blood samples from carriers of tumors with *KRAS, NRAS*, or *BRAF* mutations were also analyzed by digital droplet PCR commercial assay for the QX200 system (Biorad).

All PCR amplifications were performed in duplicate.

### Data Collection

The following data were collected from medical records: patient characteristics, PS, presence of tumor *in-situ*, number and sites of metastases, mutational status of *KRAS, NRAS*, and *BRAF*, amplification of *HER2*, and MSI status, baseline lactate dehydrogenase (LDH), carcinoembryonic antigen (CEA).

Tumor response was evaluated according to Response Evaluation Criteria in Solid Tumors (RECIST) version 1.1. CT scan was performed every 8 weeks from treatment start. PFS was defined as the time from initiation of regorafenib treatment to either radiological or clinical disease progression or death from any reason.

OS was defined as the time from initiation of regorafenib treatment to death from any reason. The cut-off for analyses and follow-up for survival status was 1st March 2018.

Information about regorafenib associated toxicities and dose reduction can be found in Supplementary Table S1 and are summarized in Supplementary Table S2.

### Statistical Analysis

All analyses were carried out using the STATA (22) and R software (23). Univariate and multivariate analysis were carried out in all evaluable patients (Figure 1). Hazard ratios and 95% CIs for PFS and OS were calculated using the stratified Cox model. Kaplan–Meier curves and comparison were computed using GraphPad 7 (Prism). All other analyses were descriptive. All *P*-values are two-sided.

## Results

Patient characteristics are summarized in Table 1. A total of 39 patients were male, and the median age was 60 years old. A majority of patients exhibited a good PS (57% with ECOG PS = 0). The median number of previous chemotherapy line was two (range from one to seven). The median number of metastatic sites was three, involving in the majority of cases liver, lung and lymph nodes. Eighty three percent of patients (*n* = 63) had prior resection of the primary tumor. Tumor molecular profiling was retrieved from clinical documentation when available: 62% (*n* = 44/71) were *KRAS* mutant, 3% (*n* = 2/58) *NRAS* mutant, 4% (*n* = 3/68) *BRAF* mutant and 20% (*n* = 6/30) HER2 amplified.

**Table 1:** Patient characteristics.

**Characteristic**	**(*n = 76*)**
Male gender–no. (%)	*39* (51%)
**Age–year**	
Median	60
Range	30–84
**WHO performance status–no. (%)**	
0	*43* (57%)
1	*33* (43%)
**Previous CT line**	
Median	2
Range	1–7
Primary tumor resected–no. (%)	*63* (83%)
**Number of metastatic sites**	
Median	3
Range	1–10
**Metastases–no. (%)**
Peritoneum	*20* (26%)
Liver	*50* (66%)
Lung^*^	*49* (65%)
Nodes	*35* (46%)
Bone§	*9* (12%)
Other§	*18* (24%)
**Molecular profile–no. (%)**	
**KRAS mutation**	
Available in	*71* (93%)
Mutated	*44* (62%)
**NRAS mutation**	
Available in	*58* (76%)
Mutated	*2* (3%)
**BRAF mutation**	
Available in	*68* (89%)
Mutated	*3* (4%)
**Her 2 amplification**	
Available in	*30* (39%)
Mutated	*6* (20%)
**MSI**	
Available in	*44* (58%)
Mutated	*4* (9%)
**OS–months**	
Median	5
Range	1–52
**PFS–weeks**	
Median	10
Range	3–52
Alive–no. (%)	*19* (25%)

* 75 observations;

§ *74 observations*.

In this cohort no partial response was seen according to RECIST criteria, and the best response was stable disease (SD) in 32.9% (*n* = 25/76) of patients. At a median follow-up time of 5.5 months (1.25–56.5 months), median OS was 5.0 months (range 1–52 months) and median PFS was 10 weeks (range 3–52 weeks).

Main toxicities upon treatment were hand-foot syndrome (46%), hypertension (27%) and skin rash (22%) (summarized data are reported in Supplementary Table S2). Dose reduction was required in 41 patients (55%) and 10 patients stopped treatment due to toxicities (13%).

Sixty five patients (86%) had blood draws available for subsequent cfDNA analyses. Fifty-seven patients had both baseline and on-treatment samples, while three cases only had baseline plasma and five patients only under treatment.

### Circulating DNA Markers at Baseline

**Figure 2:**
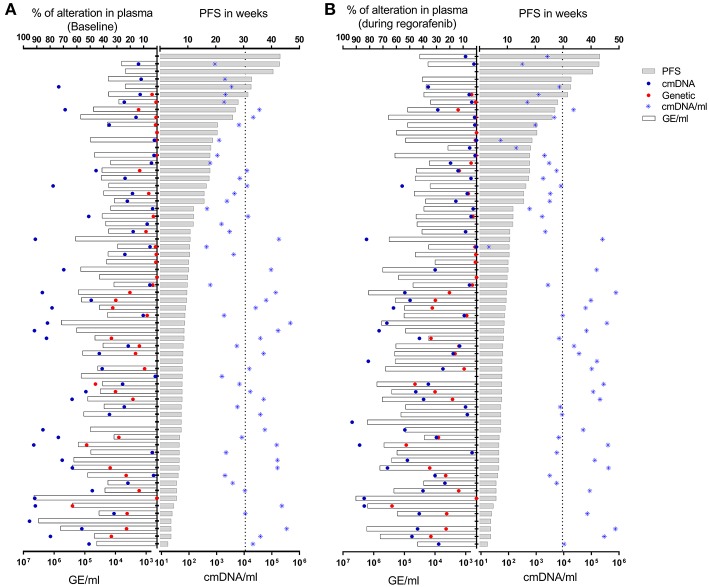
Total amount of cfDNA (expressed as GE/ml), absolute percentage of mutant (genetic) or methylated (cmDNA) alleles, normalized fraction of methylated cfDNA (cmDNA/ml) in plasma samples drawn at baseline **(A)** or under regorafenib treatment **(B)**. Patients are stratified according to their PFS. Vertical dotted lines correspond to median value for cmDNA/ml for each time point.

**Figure 3:**
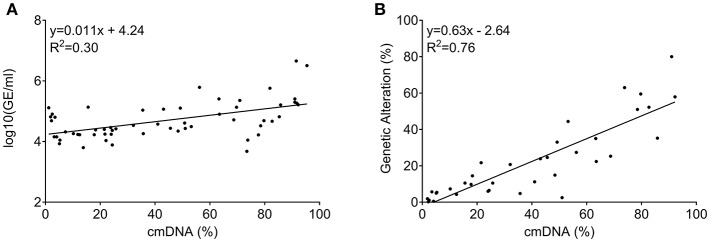
Correlation among circulating DNA markers measured at baseline (prior to regorafenib treatment). **(A)** Correlation between total amount of circulating DNA (measured as GE/ml) and cmDNA. **(B)** Correlation between cancer specific genetic alteration levels (RAS/BRAF mutant alleles) and cmDNA when both were positive.

Quantity of total cfDNA was successfully determined in all 60 patients from whom baseline LB was available. cfDNA concentration ranged from 4,750 to 4,541,672 genome equivalents per milliliter (GE/ml). Assessment of cmDNA was successful in all samples and positivity was observed in 95% (*n* = 57/60). In the three negative samples (5%), the assay was unable to detect any methylation signal above the limit of detection or limit of blank (Figure 2A). This suggests a lack of sensitivity of the assay, possibly due to very limited DNA release from these specific tumors or technical issues related to the DNA extraction process. Another explanation could be the specific site of metastases in those patients. In two out of the three cmDNA negative cases, metastases were limited to lung and lymph node or lung only. A correlation was observed between the cmDNA fraction and the total amount of circulating DNA (expressed in log(GE/ml) in baseline samples: 0.54 (95%CI: [0.33–0.70]; *p* = 1.24e-05; Figure 3A).

Forty one patients with baseline LB sample presented archival tumor tissue mutated for *KRAS, NRAS*, or *BRAF* (68.3%), and *38/41* (92.6%) demonstrated concordant status for the expected patient specific mutations, as supported by more than one mutational event out of two replicates (Figure 2A). Of note, out of the 3/41 LB samples found to be negative for mutations, two were instead positive for cmDNA, while the remaining case was negative for both genetic and epigenetic alterations. The cmDNA fraction was informative in all *19* RAS/BRAF wild type patients, therefore bypassing the need to extensively sequence cancer tissue and design individualized assays in this subpopulation.

Concordance between positive genetic allelic-ratio and cmDNA for baseline samples was 0.87 (95%CI: [0.76–0.93]; *p* = 6.2e-12; Figure 3B), demonstrating the interchangeability of both marker types when both are informative.

### Circulating DNA Markers During Treatment and Their Dynamics

Sixty two blood samples drawn after regorafenib start were available (median blood draw time: 14 days (6–75).

Quantity of total cfDNA could be determined in all 62 samples and ranged from 3,764 to 3,810,759 GE/ml. Assessment of cmDNA was successful in all samples and positivity was observed in 90% (*n* = 56/62). Forty patients presented mutated tumor (66.7%) and *38/42* (90%) demonstrated positivity in the on-treatment blood draw (Figure 2B).

We previously observed that changes in circulating DNA markers during chemotherapy treatment were shown to be correlated with patient outcome (21). We therefore investigated the relation between cfDNA markers and outcome upon regorafenib treatment by comparing cfDNA values at baseline (before treatment) and at the first blood draw (day 6–75, median: 14) after treatment initiation. Fifty-two patients had blood draw for both time points.

**Figure 4:**
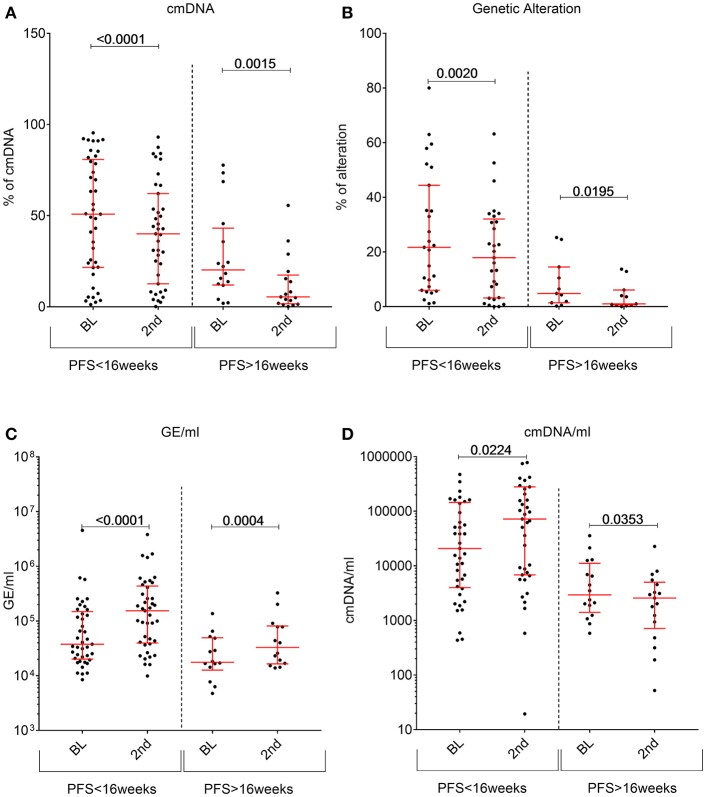
Changes in circulating DNA features between baseline plasma samples (abbreviated as BL) and the second plasma time point collected during regorafenib treatment (labeled as 2nd). For each feature patients were stratified according to their PFS status at 16 weeks. **(A)** cmDNA, **(B)** Genetic alterations, **(C)** total cfDNA amount in GE/ml, **(D)** cmDNA/ml. *p*-values compiled using two-tailed *u*-test.

Contrary to what we previously observed with other drug regimens, regorafenib induced a significant decrease in cancer specific markers (genetic or cmDNA; Figures 4A,B) in most patients regardless of the response status, while total amount of cfDNA (measured by GE/ml) usually increased upon treatment (Figure 4C). In order to shed light on this unexpected finding, we tested whether regorafenib was able to differentially and directly modulate active release of cfDNA from normal or malignant cells. However, *in-vitro* treatment of either non-malignant or cancer cell lines showed that regorafenib did not significantly affect cfDNA release (data not shown). One alternative hypothesis is that the total amount of cfDNA could originate mostly from non-neoplastic cells due to drug on target and off-target broad effects on several cell types, tissues, and organs. Partially supporting this hypothesis, we observed a non-significant increase in total cfDNA amount (GE/ml) change in patients who required a dose reduction (due to toxicities) while the cmDNA (expected to be solely of tumor origin) remained similar between the two subgroups (Supplementary Figure 1). Therefore, we reasoned that the cmDNA fraction should be normalized to total amounts of cfDNA by taking into account the GE/ml, resulting in cmDNA/ml (calculated by multiplying the cmDNA by GE/ml). Compared to baseline, we found that this parameter significantly increases under treatment in patients with fast relapse while it significantly decrease under treatment in patients achieving ≥16 weeks disease control with regorafenib (Figure 4D).

**Figure 5:**
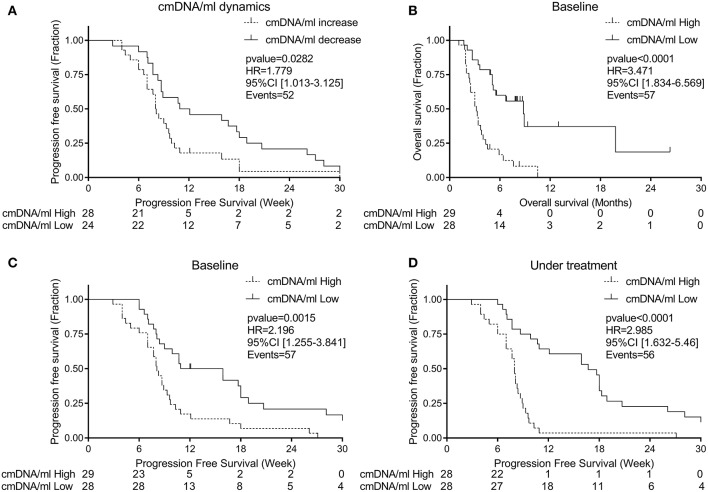
**(A)** Progression free survival according to cmDNA/ml dynamics (decrease dash line; increase solid line), **(B)** Overall survival according to baseline cmDNA/ml, **(C)** Progression free survival according to baseline cmDNA/ml, **(D)** Progression free survival according to level of cmDNA/ml under treatment (Low ctDNA level dash line; High ctDNA solid line).

In a Cox regression model, we confirmed that an increase in cmDNA/ml from baseline was associated with worst PFS (Figure 5A) (*p* = 0.028, HR = 1.78 [95% CI: 1.01–3.13]).

### Survival Analysis for Clinico-Pathological Characteristics and Circulating DNA Markers

In the univariate analysis, factors significantly associated with shorter PFS were a high cmDNA/ml fraction at baseline (*p* = 7.73e-06), baseline LDH (*p* = 0.0143), and regorafenib dose reduction (*p* = 0.036).

There was no statistically significant association between PFS and either ECOG PS (*p* = 0.11) or the presence of primary tumor (*p* = 0.11) or drug-related toxicity. Age, previous CT line, baseline CEA and peritoneal metastasis were not significantly associated with PFS (*p* = 0.43, *p* = 0.92, *p* = 0.42, and *p* = 0.85, respectively). In the multivariate analysis, adjusted on baseline LDH, the amount of cmDNA/ml (as log transformed) at baseline remained significant (HR: 1.50 [95%CI: 1.23–1.82], *p* = 6.03e-05).

By univariate analysis, high cmDNA/ml at baseline (*p* = 3.61e-08) and increased bilirubin (*p* = 0.003) were associated with worst OS, while presence of hand foot syndrome associated with better OS (*p* = 0.019). There was no statistically significant association between OS and baseline CEA (*p* = 0.117), baseline LDH (*p* = 0.079), presence of primary tumor (*p* = 0.106), peritoneal metastasis (*p* = 0.186), and regorafenib dose reduction (*p* = 0.083). Age, previous CT line and ECOG PS were not significantly associated with OS (*p* = 0.43, *p* = 0.84, *p* = 0.96, respectively). In the multivariate model, the log transformed cmDNA/ml at baseline remained statistically significant (HR = 1.53 [95%CI: 1.25–1.87], *p* = 2.72e-05).

We performed in parallel a Cox-regression for OS using median as a cut-off value for baseline, and we found an association between high cmDNA/ml at baseline and worst survival (Figure 5B; HR for death = 3.471, *p* < 0.0001, [95% CI: 1.83–6.57]). Since in mCRC PFS is a surrogate endpoint for OS (24), further analyses were conducted to explore differences in terms of PFS. Using median as cut-off on both time points, we found an association between higher risk of relapse during regorafenib treatment and high cmDNA/ml at baseline (Figure 5C; HR for progression = 2.196, *p* = 0.0015, [95% CI: 1.26–3.84]) and during treatment (Figure 5D; HR for progression = 2.985, *p* < 0.0001, [95% CI: 1.63–5.46]).

## Discussion

Due to its stability and cancer specificity, cmDNA could be a promising source for tumor biomarkers. Recent studies analyzed the correlation between the methylation status of specific genes and response to therapy and prognosis in colon and rectal cancers (25–27).

In the present study, we have shown that by using a universal five-gene panel, cmDNA was detectable in 57/60 cases, corresponding to 95% of the whole patient population. In contrast, in the same cohort through the analysis of candidate hotspot mutations in RAS/BRAF genes by LB, we were able to detect mutations in plasma cfDNA in only 38/60 patients, corresponding to 95% of cases known to carry RAS/BRAF mutations in tumor tissue samples, but only to 63.3% of the entire cohort. This confirmed the universality of methylation markers for liquid biopsy and their possible better suitability for large cohort analyses such as epidemiologic studies.

To overcome the caveats and costs of assessing genetic alterations in cfDNA by large panels, several studies have proposed the use of targeting sequencing in archival tissue to identify cancer patient-specific genetic markers (12, 28–30). While such approach can certainly improve the specificity of LB assays, it would undoubtedly increase the costs as well as the sample processing time (due to optimization of single variation assay). This is of clinical importance since absence of candidate mutation would require NGS analysis of primary tumor and subsequent personal assay design, impairing the clinical turnaround, and the application of cfDNA in prospective analyses. Consequently, methylation assays might be more prone to be developed into routine clinically applicable tests for disease monitoring purposes. In this regard, we note that methylation based assays of cfDNA have already been proposed for early detection and cancer classification (31, 32).

We and others demonstrated that cmDNA reflects tumor burden; since its level correlated with the presence of unresected primary tumor, of bulky disease or of multiple metastatic lesions, whereas age and mutational status did not influence the cmDNA (21, 33, 34). We speculated that longitudinal assessment of cmDNA during chemotherapy could reflect the dynamics of tumor burden with a decrease potentially preceding response and an increase anticipating progression.

In the 52 mCRC patients with both evaluable baseline and under treatment plasma samples from present study, we observed a decline in cancer specific cfDNA markers (genetic or cmDNA) upon treatment with regorafenib that unexpectedly took place in most cases, while total amount of cfDNA increased. This behavior of cancer specific markers was different from what we previously observed using other anticancer therapies (5FU based chemotherapy, panitumumab or temozolomide). We therefore speculate that the observed decrease in cancer specific markers during regorafenib treatment could be due to a diluting effect by normal DNA shedding from healthy tissues (possibly due to cytotoxicity) as previously suggested by a report on a cohort of 20 regorafenib treated mCRC patients (12). This observation highlights the need for careful validation of LB assay according to the treatment used, and warrants fundamental research to improve our understanding of the factors influencing release of cfDNA by human cells.

Nevertheless, after normalization, a cmDNA/ml an increase upon treatment was associated with progressive disease while a decrease was associated with clinical benefit and improved PFS showing that despite being confounded by normal DNA release, dynamics of cmDNA was associated with drug activity. Contrary to conventional chemotherapy or targeted therapy based regimens which often trigger significant tumor burden changes, regorafenib seldom induces RECIST responses. The lack of tumor burden dynamics given by regorafenib is likely to influence the dynamics of ctDNA levels.

We identified cmDNA/ml at baseline as a prognostic marker. Through a multivariate cox-regression analysis, cmDNA/ml maintained a significant impact on both PFS and OS which was higher than other clinical variables such as age, previous CT, ECOG PS, presence of primary tumor, peritoneal metastases, CEA, and LDH. Consequently, integration of cmDNA/ml as auxiliary staging parameter might improve patient disease classification (35).

High values of cmDNA/ml (above median) during treatment were significantly associated with a higher probability for disease progression, suggesting that abrogation of ctDNA release needs to be achieved soon after treatment initiation in order to observe better PFS. This is in accordance with the literature demonstrating that early circulating biomarkers change is associated to clinical benefit (10, 12).

We acknowledge that our study is limited by its retrospective nature. Recent studies (36, 37) suggested that toxicity might be related to better prognosis upon regorafenib treatment. In our dataset, no specific toxicity was associated with improved PFS, however the need for dose reduction (surrogate for toxicity related comorbidity) was associated with improved response duration.

We found a difference in cmDNA/ml levels between progressing patients and those who achieved clinical benefit. Nevertheless, the establishment of an optimized threshold to clearly distinguish between these populations will be needed to stratify individual patients. Unfortunately, the number of patients with clinical benefit was relatively small due to the modest efficacy shown by regorafenib in this setting. Therefore, modeling an optimal threshold in our cohort was not possible due to the lack of statistical power. As a consequence, we preferred to use the median as a natural and unbiased cut-off for this work. We acknowledge that our study could not provide validation of such a cut-off in a separate cohort. Future efforts in this direction will require multicenter enrollment and long term follow-up to reach a large number of cases with clinical benefit. This may validate the application of cmDNA/ml for predicting regorafenib response in individual patients. Nevertheless, this is the first report investigating the correlation between survival and a methylated gene panel in mCRC treated with regorafenib. Furthermore, data from the present cohort confirmed the general predictive value of cmDNA in mCRC reported in our previous work (21). However, the dynamics of the cmDNA may be affected by different treatments, since we observed a peculiar decrease in cancer specific markers (genetic or cmDNA) during regorafenib treatment in most of patients regardless of the response status warranting its normalization by total amount of cfDNA.

In conclusion, cmDNA/ml is of prognostic value and is a dynamic biomarker which longitudinal assessment could be used relatively early during the treatment of mCRC patients, before radiological assessment, to identify the patients with a negative prognosis.

## Conclusions

To conclude, DNA methylation in cfDNA is a cancer specific biomarker that could be employed to track response during therapy in mCRC, enabling non-invasive monitoring of tumor burden. It could be used to select patients with poor survival who are not likely to benefit from regorafenib treatment and might allow faster therapeutic reorientation avoiding overexposure to the drug and possible side effects.

## Ethics Statement

This study was carried out in accordance with the recommendations of Niguarda Cancer Center (Milano) and Istituto Oncologico Veneto (Padova) Institutional Ethics Committee with written informed consent from all subjects. All subjects gave written informed consent in accordance with the Declaration of Helsinki. The protocol was approved by the Niguarda Cancer Center (Milano) and Istituto Oncologico Veneto (Padova) Institutional Ethics Committee.

## Author Contributions

AA, MS, FT, SL, EB, LPa, DP, EP, FB, GA, RI, LPr, VZ, SS, FL, and AS-B participated in the patients recruitment and follow-up and in the acquisition of clinical information and blood draws. LB and BM processed the blood samples (cfDNA extraction) and performed the molecular experiments. AA, MS, FT, and LB reviewed the data. MN, AA, and LB performed the statistical analyses. MA provided preclinical samples and participated in functional analyses design. AA, MA, MS, SM, VZ, SS, AB, FL, FD, AS-B, and LB designed the study. FD, AB, and LB designed the molecular experiments. AA, MS, FD, AS-B, and LB wrote the manuscript. All authors revised the manuscript critically for important intellectual content and approved its final version.

### Conflict of Interest Statement

AA participated to advisory boards for Amgen and Bayer. AS-B participated in advisory boards for Amgen, Bayer, and Sanofi. The remaining authors declare that the research was conducted in the absence of any commercial or financial relationships that could be construed as a potential conflict of interest. The reviewer RG declared a past co-authorship with several of the authors MS, FL, FB, SL, and VZ to the handling editor.

## Abbreviations

CEA: carcinoembryonic antigen
cfDNA: cell free circulating DNA
ctDNA: cfDNA of tumor origin
cmDNA: circulating methylated DNA
CRC: colorectal cancer
HR: hazard ratio
LB: liquid biopsy
LDH: lactate dehydrogenase
mCRC: metastatic CRC
OR: odds ratio
OS: overall survival
PFS: Progression free survival
RECIST: Response Evaluation Criteria in Solid Tumors.

## References

[1] Siegel RL Miller KD Jemal A Cancer statistics, 2018. CA Cancer J Clin. (2018) 68:7–30. 10.3322/caac.21442 29313949

[2] Heinemann V Von Weikersthal LF Decker T Kiani A Vehling-Kaiser U Al-Batran SE. FOLFIRI plus cetuximab versus FOLFIRI plus bevacizumab as first-line treatment for patients with metastatic colorectal cancer (FIRE-3): a randomised, open-label, phase 3 trial. Lancet Oncol. (2014) 15:1065–75. 10.1016/S1470-2045(14)70330-4 25088940

[3] Loupakis F Cremolini C Masi G Lonardi S Zagonel V Salvatore L. Initial therapy with FOLFOXIRI and bevacizumab for metastatic colorectal cancer. N Engl J Med. (2014) 371:1609–18. 10.1056/NEJMoa1403108 25337750

[4] Abou-Elkacem L Arns S Brix G Gremse F Zopf D Kiessling F. Regorafenib inhibits growth, angiogenesis, and metastasis in a highly aggressive, orthotopic colon cancer model. Mol Cancer Ther. (2013) 12:1322–31. 10.1158/1535-7163.MCT-12-1162 23619301

[5] Schmieder R Hoffmann J Becker M Bhargava A Muller T Kahmann N. Regorafenib (BAY 73-4506): antitumor and antimetastatic activities in preclinical models of colorectal cancer. Int J Cancer. (2014) 135:1487–96. 10.1002/ijc.28669 24347491 PMC4277327

[6] Strumberg D Schultheis B. Regorafenib for cancer. Expert Opin Investig Drugs. (2012) 21:879–89. 10.1517/13543784.2012.684752 22577890

[7] Grothey A Van Cutsem E Sobrero A Siena S Falcone A Ychou M. Regorafenib monotherapy for previously treated metastatic colorectal cancer (CORRECT): an international, multicentre, randomised, placebo-controlled, phase 3 trial. Lancet. (2013) 381:303–12. 10.1016/S0140-6736(12)61900-X 23177514

[8] Li J Qin S Xu R Yau TC Ma B Pan H. Regorafenib plus best supportive care versus placebo plus best supportive care in Asian patients with previously treated metastatic colorectal cancer (CONCUR): a randomised, double-blind, placebo-controlled, phase 3 trial. Lancet Oncol. (2015) 16:619–29. 10.1016/S1470-2045(15)70156-7 25981818

[9] Tabernero J Lenz HJ Siena S Sobrero A Falcone A Ychou M. Analysis of circulating DNA and protein biomarkers to predict the clinical activity of regorafenib and assess prognosis in patients with metastatic colorectal cancer: a retrospective, exploratory analysis of the CORRECT trial. Lancet Oncol. (2015) 16:937–48. 10.1016/S1470-2045(15)00138-2 26184520 PMC7513622

[10] Komori A Taniguchi H Hamauchi S Masuishi T Kito Y Narita Y. Serum CA19-9 response is an early predictive marker of efficacy of regorafenib in refractory metastatic colorectal cancer. Oncology. (2017) 93:329–35. 10.1159/000479280 28866662

[11] Khan K Rata M Cunningham D Koh DM Tunariu N Hahne JC. Functional imaging and circulating biomarkers of response to regorafenib in treatment-refractory metastatic colorectal cancer patients in a prospective phase II study. Gut. (2018) 67:1484–92. 10.1136/gutjnl-2017-314178 28790159 PMC6204951

[12] Vandeputte C Kehagias P El Housni H Ameye L Laes JF Desmedt C. Circulating tumor DNA in early response assessment and monitoring of advanced colorectal cancer treated with a multi-kinase inhibitor. Oncotarget. (2018) 9:17756–69. 10.18632/oncotarget.24879 29707145 PMC5915153

[13] Warren JD Xiong W Bunker AM Vaughn CP Furtado LV Roberts WL. Septin 9 methylated DNA is a sensitive and specific blood test for colorectal cancer. BMC Med. (2011) 9:133. 10.1186/1741-7015-9-133 22168215 PMC3271041

[14] Lange CP Campan M Hinoue T Schmitz RF Van Der Meulen-De Jong AE Slingerland H. Genome-scale discovery of DNA-methylation biomarkers for blood-based detection of colorectal cancer. PLoS ONE. (2012) 7:e50266. 10.1371/journal.pone.0050266 23209692 PMC3508917

[15] Philipp AB Stieber P Nagel D Neumann J Spelsberg F Jung A. Prognostic role of methylated free circulating DNA in colorectal cancer. Int J Cancer. (2012) 131:2308–19. 10.1002/ijc.27505 22362391

[16] Roperch JP Incitti R Forbin S Bard F Mansour H Mesli F. Aberrant methylation of NPY, PENK, and WIF1 as a promising marker for blood-based diagnosis of colorectal cancer. BMC Cancer. (2013) 13:566. 10.1186/1471-2407-13-566 24289328 PMC4219483

[17] Church TR Wandell M Lofton-Day C Mongin SJ Burger M Payne SR. Prospective evaluation of methylated SEPT9 in plasma for detection of asymptomatic colorectal cancer. Gut. (2014) 63:317–25. 10.1136/gutjnl-2012-304149 23408352 PMC3913123

[18] Hao TB Shi W Shen XJ Qi J Wu XH Wu Y. Circulating cell-free DNA in serum as a biomarker for diagnosis and prognostic prediction of colorectal cancer. Br J Cancer. (2014) 111:1482–9. 10.1038/bjc.2014.470 25157833 PMC4200099

[19] Okugawa Y Grady WM Goel A. Epigenetic alterations in colorectal cancer: emerging biomarkers. Gastroenterology. (2015) 149:1204–25 e1212. 10.1053/j.gastro.2015.07.011 26216839 PMC4589488

[20] Bartak BK Kalmar A Peterfia B Patai AV Galamb O Valcz G. Colorectal adenoma and cancer detection based on altered methylation pattern of SFRP1, SFRP2, SDC2, and PRIMA1 in plasma samples. Epigenetics. (2017) 12:751–63. 10.1080/15592294.2017.1356957 28753106 PMC5739100

[21] Barault L Amatu A Siravegna G Ponzetti A Moran S Cassingena A. Discovery of methylated circulating DNA biomarkers for comprehensive non-invasive monitoring of treatment response in metastatic colorectal cancer. Gut. (2018) 67:1995–2005. 10.1136/gutjnl-2016-313372 28982739 PMC5897187

[22] Statacorp (2017). Stata Statistical Software: Release 15. College Station, TX: StataCorp LLC.

[23] R_Core_Team (2013). R: A Language and Environment for Statistical Computing. Vienna: R Foundation for Statistical Computing.

[24] Buyse M Burzykowski T Carroll K Michiels S Sargent DJ Miller LL. Progression-free survival is a surrogate for survival in advanced colorectal cancer. J Clin Oncol. (2007) 25:5218–24. 10.1200/JCO.2007.11.8836 18024867

[25] Ebert MP Tanzer M Balluff B Burgermeister E Kretzschmar AK Hughes DJ. TFAP2E-DKK4 and chemoresistance in colorectal cancer. N Engl J Med. (2012) 366:44–53. 10.1056/NEJMoa1009473 22216841

[26] Barault L Amatu A Bleeker FE Moutinho C Falcomata C Fiano V. Digital PCR quantification of MGMT methylation refines prediction of clinical benefit from alkylating agents in glioblastoma and metastatic colorectal cancer. Ann Oncol. (2015) 26:1994–9. 10.1093/annonc/mdv272 26113646

[27] Herbst A Vdovin N Gacesa S Ofner A Philipp A Nagel D. Methylated free-circulating HPP1 DNA is an early response marker in patients with metastatic colorectal cancer. Int J Cancer. (2017) 140:2134–44. 10.1002/ijc.30625 28124380

[28] Tie J Kinde I Wang Y Wong HL Roebert J Christie M. Circulating tumor DNA as an early marker of therapeutic response in patients with metastatic colorectal cancer. Ann Oncol. (2015) 26:1715–22. 10.1093/annonc/mdv177 25851626 PMC4511218

[29] Guo Q Wang J Xiao J Wang L Hu X Yu W. Heterogeneous mutation pattern in tumor tissue and circulating tumor DNA warrants parallel NGS panel testing. Mol Cancer. (2018) 17:131. 10.1186/s12943-018-0875-0 30153823 PMC6114875

[30] Yamauchi M Urabe Y Ono A Miki D Ochi H Chayama K. Serial profiling of circulating tumor DNA for optimization of anti-VEGF chemotherapy in metastatic colorectal cancer patients. Int J Cancer. (2018) 142:1418–26. 10.1002/ijc.31154 29134647

[31] Liu L Toung JM Jassowicz AF Vijayaraghavan R Kang H Zhang R. Targeted methylation sequencing of plasma cell-free DNA for cancer detection and classification. Ann Oncol. (2018) 29:1445–53. 10.1093/annonc/mdy119 29635542 PMC6005020

[32] Shen SY Singhania R Fehringer G Chakravarthy A Roehrl MHA Chadwick D. Sensitive tumour detection and classification using plasma cell-free DNA methylomes. Nature. (2018) 563:579–83. 10.1038/s41586-018-0703-0 30429608

[33] Garrigou S Perkins G Garlan F Normand C Didelot A Le Corre D. A study of hypermethylated circulating tumor DNA as a universal colorectal cancer biomarker. Clin Chem. (2016) 62:1129–39. 10.1373/clinchem.2015.253609 27251038

[34] Bhangu JS Beer A Mittlbock M Tamandl D Pulverer W Schonthaler S. Circulating free methylated tumor DNA markers for sensitive assessment of tumor burden and early response monitoring in patients receiving systemic chemotherapy for colorectal cancer liver metastasis. Ann Surg. (2018) 268:894–902. 10.1097/SLA.0000000000002901 30080722

[35] Bergheim J Semaan A Gevensleben H Groening S Knoblich A Dietrich J. Potential of quantitative SEPT9 and SHOX2 methylation in plasmatic circulating cell-free DNA as auxiliary staging parameter in colorectal cancer: a prospective observational cohort study. Br J Cancer. (2018) 118:1217–28. 10.1038/s41416-018-0035-8 29610456 PMC5943265

[36] Giampieri R Prete MD Prochilo T Puzzoni M Pusceddu V Pani F. Off-target effects and clinical outcome in metastatic colorectal cancer patients receiving regorafenib: the TRIBUTE analysis. Sci Rep. (2017) 7:45703. 10.1038/srep45703 28378839 PMC5380985

[37] Schirripa M Pasqualetti G Giampieri R Scartozzi M Lonardi S Rumano L. Prognostic value of thyroid hormone ratios in patients with advanced metastatic colorectal cancer treated with regorafenib: the TOREADOR study. Clin Colorectal Cancer. (2018) 17:e601–15. 10.1016/j.clcc.2018.05.013 30149875

