# Supplementary material for: High Circulating Methylated DNA Is a Negative Predictive and Prognostic Marker in Metastatic Colorectal Cancer Patients Treated With Regorafenib
Source: Front Oncol. 2019 Jul 12;9:622. doi: 10.3389/fonc.2019.00622 (PMC6640154; doi:10.3389/fonc.2019.00622)
Supplement: Supplementary file 1 [file Table_1.pdf]

**Supplementary Table:**

Summary of main adverse events in patients evaluable for toxicity (n=76), who received at least first cycle of regorafenib treatment.

The table shows also frequency, reason and timing of dose reductions.

| **Toxicities** | **G1-G2** *n* (%) | **G3-G4** *n* (%) |
|---|---|---|
| Hypertension | 11 (14%) | 10 (13%) |
| Skin rash | 10 (13%) | 7 (9%) |
| Hand-foot syndrome | 20 (26%) | 15 (20%) |
| Mucositis | 16 (21%) | 4 (5%) |
| Diarrhea | 8 (10%) | 3 (4%) |
| Bilirubin increase | 16 (21%) | 1 (1%) |
| AST/ALT increase | 13 (17%) | 1 (1%) |
| Thrombocytopenia | 8 (10%) | 0 |
| Anemia | 7 (9%) | 0 |
| Other toxicities | 37 (49%) | 14 (18%) |
| **Dose reduction** | 41/74 (55%) | |
| **Dose reduction -1 dose level** | 29/41 (70%) | |
| **Dose reduction -2 dose level** | 12/41 (30%) | |
| **Reason for dose reduction** | | |
| Hypertension | 1/41 (2%) | |
| Skin rash / HFS | 20/41 (49%) | |
| Mucositis | 2/41 (5%) | |
| Diarrhea | 2/41 (5%) | |
| Other | 16/41 (39%) | |
| **Dose reduction occurs** | | |
| During first cycle | 18/41 (44%) | |
| At second cycle | 12/41 (29%) | |
| At third cycle | 5/41 (12%) | |
| After fourth cycle | 6/41 (15%) | |
| **Stop for toxicities** | 10/76 (13%) | |
