# Supplementary material for: High Circulating Methylated DNA Is a Negative Predictive and Prognostic Marker in Metastatic Colorectal Cancer Patients Treated With Regorafenib
Source: Front Oncol. 2019 Jul 12;9:622. doi: 10.3389/fonc.2019.00622 (PMC6640154; doi:10.3389/fonc.2019.00622)
Supplement: Supplementary file 3 [file Image_1.pdf]

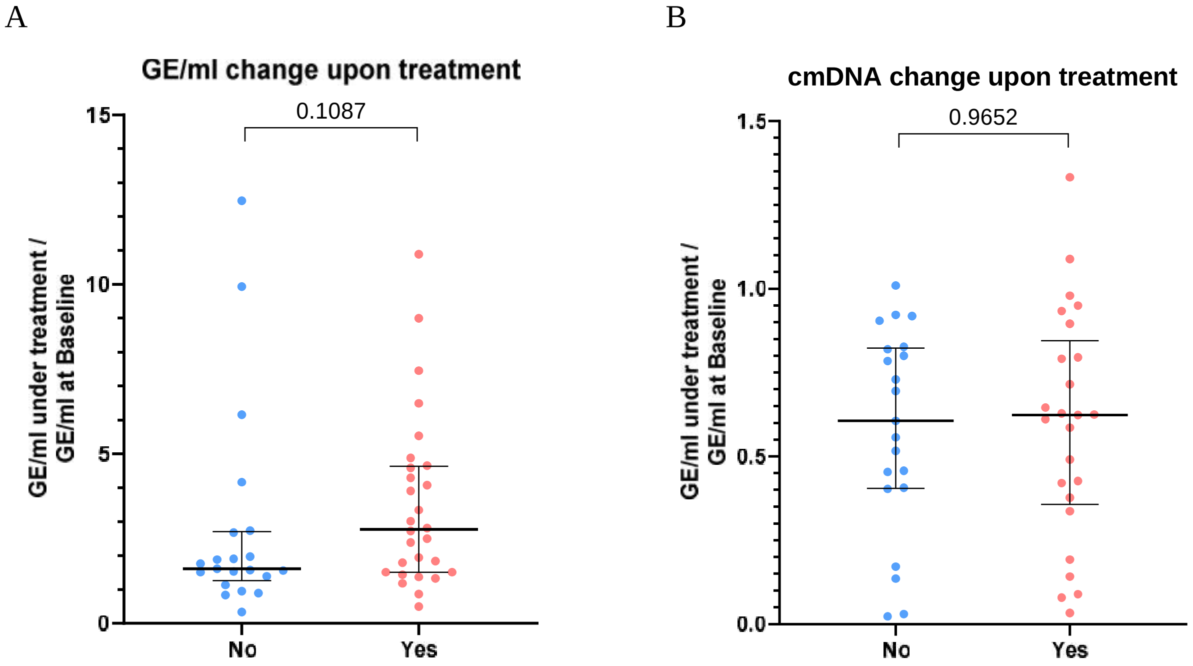

Supplemental Figure 1: Change of GE/ml (total amount of circulating DNA) [A] or cmDNA (circulating DNA of tumor origin) [B], between under treatment and baseline timepoint. Stratification of the patients is performed according to the need for dose reduction (Yes/No). While a non-significant increase in total cfDNA amount (GE/ml) change in patients who required a dose reduction is observed the cmDNA (expected to be solely of tumor origin) remained similar between the two subgroups.
