# Supplementary material for: High Circulating Methylated DNA Is a Negative Predictive and Prognostic Marker in Metastatic Colorectal Cancer Patients Treated With Regorafenib
Source: Front Oncol. 2019 Jul 12;9:622. doi: 10.3389/fonc.2019.00622 (PMC6640154; doi:10.3389/fonc.2019.00622)
Supplement: Supplementary file 2 [file Data_Sheet_1.PDF]

	CLINICAL FEATURES													
Patitent_ID	Sex (Male = 1)	Age	Previous CT regimen	REGO_START_DATE	BASELINE_SUM_RECIST	BASELINE_SCAN_DATE	BULKY_DISEASE	N_TARGET_LESIONS	T_IN_SITU	N_METASTATIC_SITES	LIVER	LUNG	NODES	PERITONEUM
REGO_study_Pat_1	1	59	2	10/15/2015	36	10/2/2015	0	2	0	1	1	0	0	1
REGO_study_Pat_2	0	44	2	2/26/2016	108	2/10/2016	1	2	1	2	1	1	0	0
REGO_study_Pat_3	0	60	3	9/3/2015	164	8/6/2015	0	4	0	10	0	0	1	0
REGO_study_Pat_4	0	63	2	9/17/2015	35	8/17/2015	0	1	0	1	1	0	0	0
REGO_study_Pat_5	1	80	4	9/21/2016	24	8/26/2016	0	1	0	5	0	0	1	0
REGO_study_Pat_6	1	72	4	10/21/2015	20	9/9/2015	0	1	0	3	1	1	1	0
REGO_study_Pat_7	0	67	2	4/27/2016	87	4/7/2016	0	3	1	3	1	0	0	1
REGO_study_Pat_8	1	59	3	3/31/2016	56	3/15/2016	0	2	0	3	0	1	1	1
REGO_study_Pat_9	1	78	3	4/15/2016	85	3/25/2016	0	2	0	1	1	0	0	0
REGO_study_Pat_10	1	76	2	9/17/2015	70	7/28/2015	0	1	1	1	1	0	0	0
REGO_study_Pat_11	1	36	2	9/16/2015	26	8/27/2015	0	1	1	3	1	1	1	0
REGO_study_Pat_12	0	73	2	12/3/2015	NA	12/3/2015	0	NA	0	NA	1	NA	1	1
REGO_study_Pat_13	0	56	2	10/1/2015	39	9/3/2015	0	2	0	1	0	1	0	0
REGO_study_Pat_14	1	57	2	12/10/2015	10	11/6/2015	0	1	1	3	0	1	1	1
REGO_study_Pat_15	1	84	3	2/16/2017	41	1/16/2017	0	2	0	2	1	1	0	0
REGO_study_Pat_16	0	55	2	1/19/2017	10	12/20/2016	0	1	0	3	1	1	1	0
REGO_study_Pat_17	0	60	2	12/28/2016	27	12/20/2016	0	2	0	NA	1	1	0	0
REGO_study_Pat_18	1	51	2	2/9/2017	47	12/29/2016	0	2	0	2	0	1	1	0
REGO_study_Pat_19	1	44	5	6/6/2017	87	5/26/2017	0	3	0	5	1	1	1	1
REGO_study_Pat_20	0	30	3	8/24/2017	192	7/28/2017	1	3	1	3	1	1	0	0
REGO_study_Pat_21	1	50	3	8/23/2017	40	8/9/2017	0	2	0	2	0	1	0	0
REGO_study_Pat_22	1	70	3	11/11/2015	58	10/2/2015	0	2	0	1	1	0	0	0
REGO_study_Pat_23	0	66	2	9/29/2016	50	9/9/2016	0	1	1	2	0	0	0	1
REGO_study_Pat_24	0	52	2	11/11/2015	89	10/7/2015	0	2	0	5	0	1	0	1
REGO_study_Pat_25	1	71	3	1/12/2017	30	12/16/2016	0	2	0	2	0	0	0	0
REGO_study_Pat_26	1	65	2	2/8/2017	40	1/30/2017	0	2	0	4	0	1	1	0
REGO_study_Pat_27	0	45	1	6/29/2017	87	6/12/2017	0	1	0	1	0	0	0	1
REGO_study_Pat_28	1	61	2	11/30/2016	46	11/23/2016	0	2	0	3	1	0	1	1
REGO_study_Pat_29	1	70	3	10/14/2016	64	10/14/2016	0	2	0	1	0	1	0	0
REGO_study_Pat_30	0	54	1	1/3/2017	69	12/27/2016	0	3	0	4	1	1	1	1
REGO_study_Pat_31	0	72	5	3/8/2013	40	3/8/2013	0	2	0	2	0	1	1	0
REGO_study_Pat_32	0	53	5	3/26/2013	37	3/20/2013	0	2	0	3	1	1	0	0
REGO_study_Pat_33	1	53	3	2/27/2013	75	2/27/2013	0	4	0	4	1	1	1	0
REGO_study_Pat_34	1	59	3	6/21/2017	65	5/15/2017	0	1	1	2	1	1	0	0
REGO_study_Pat_35	1	57	4	10/17/2016	77	10/17/2016	0	3	0	3	1	1	1	0
REGO_study_Pat_36	1	67	3	3/7/2013	45	3/4/2013	0	2	0	2	0	1	1	0
REGO_study_Pat_37	1	73	7	11/10/2016	77	10/25/2016	0	2	0	3	1	1	0	0
REGO_study_Pat_38	0	62	3	3/19/2013	62	3/5/2013	0	2	0	3	1	1	1	0
REGO_study_Pat_39	0	56	4	11/22/2016	84	10/13/2016	1	3	0	4	1	1	1	0
REGO_study_Pat_40	0	67	2	10/5/2016	52	9/23/2016	1	2	0	1	1	0	0	0
REGO_study_Pat_41	0	64	2	2/26/2013	55	2/26/2013	0	3	0	3	0	1	1	1
REGO_study_Pat_42	1	69	4	1/12/2017	148	11/28/2016	1	2	0	3	1	1	1	0
REGO_study_Pat_43	0	47	3	12/1/2016	20	11/12/2016	0	1	0	1	0	1	0	0
REGO_study_Pat_44	0	63	5	8/31/2016	141	8/18/2016	1	1	1	3	1	1	0	1
REGO_study_Pat_45	0	69	3	1/18/2017	72	12/20/2016	1	2	0	2	1	0	1	0
REGO_study_Pat_46	1	52	3	12/4/2012	50	2/26/2013	0	2	0	3	0	1	0	0
REGO_study_Pat_47	1	73	3	10/20/2016	112	9/29/2016	0	2	0	2	1	0	1	0
REGO_study_Pat_48	1	51	2	9/1/2016	45	8/19/2016	1	1	1	1	1	0	0	0
REGO_study_Pat_49	1	62	5	6/8/2017	70	5/23/2017	0	2	0	3	1	1	0	0
REGO_study_Pat_50	0	50	2	8/31/2016	87	8/9/2016	1	2	1	3	1	1	0	0
REGO_study_Pat_51	1	61	4	10/27/2016	89	10/13/2016	1	3	0	2	1	0	0	1
REGO_study_Pat_52	0	62	4	11/24/2016	110	10/21/2016	1	2	0	3	1	1	0	1
REGO_study_Pat_53	1	50	5	5/3/2013	122	2/15/2013	0	3	1	4	1	1	1	0
REGO_study_Pat_54	0	46	5	11/22/2016	190	11/8/2016	0	3	0	3	1	1	0	0
REGO_study_Pat_55	1	60	5	9/30/2016	131	9/20/2016	0	2	0	1	0	1	0	0
REGO_study_Pat_56	0	52	3	9/22/2016	17	9/7/2016	0	1	0	2	0	1	1	0
REGO_study_Pat_57	1	65	2	8/31/2016	11	8/17/2016	0	1	0	2	0	1	0	0
REGO_study_Pat_58	1	49	3	9/27/2016	66	9/7/2016	1	2	1	2	1	0	1	0
REGO_study_Pat_59	0	58	3	2/21/2013	140	2/21/2013	1	2	0	3	1	1	1	0
REGO_study_Pat_60	0	67	3	2/26/2013	52	2/26/2013	0	1	0	3	1	0	1	0
REGO_study_Pat_61	0	76	2	8/31/2016	30	8/16/2016	0	2	0	1	0	1	0	0
REGO_study_Pat_62	1	66	2	3/8/2013	66	12/15/2013	0	1	0	2	1	1	0	0
REGO_study_Pat_63	1	71	2	9/13/2016	176	8/31/2016	0	1	0	3	0	0	1	1
REGO_study_Pat_64	0	41	2	3/6/2013	80	1/24/2013	1	1	0	1	1	0	0	0
REGO_study_Pat_65	0	60	2	3/6/2013	95	1/31/2013	0	2	0	2	1	1	0	0
REGO_study_Pat_66	0	78	3	3/28/2017	44	3/8/2017	0	1	0	3	1	1	1	0
REGO_study_Pat_67	0	64	3	9/14/2016	21	9/7/2016	0	1	0	3	0	1	1	0
REGO_study_Pat_68	0	65	3	3/7/2013	70	1/28/2013	0	2	0	2	1	0	1	0
REGO_study_Pat_69	0	49	2	3/30/2017	30	3/31/2017	0	2	0	3	0	1	1	0
REGO_study_Pat_70	0	52	5	11/3/2016	141	10/28/2016	1	3	0	3	1	1	0	1
REGO_study_Pat_71	1	83	3	1/3/2017	50	12/15/2016	0	1	0	1	1	0	0	0
REGO_study_Pat_72	1	55	6	9/27/2016	93	9/6/2016	1	1	0	2	1	0	0	1
REGO_study_Pat_73	1	62	6	8/31/2016	77	8/9/2016	0	2	0	2	0	0	1	1
REGO_study_Pat_74	1	56	6	12/15/2016	125	11/30/2016	0	1	0	2	1	1	1	0
REGO_study_Pat_75	0	64	2	4/12/2017	81	3/8/2017	1	2	0	4	1	1	0	1
REGO_study_Pat_76	1	58	2	9/1/2016	77	8/3/2016	1	2	0	1	1	0	0	0

[illegible]
